# Ultimate Scaling of High-κ Gate Dielectrics: Higher-κ or Interfacial Layer Scavenging?

**DOI:** 10.3390/ma5030478

**Published:** 2012-03-14

**Authors:** Takashi Ando

**Affiliations:** IBM Thomas J. Watson Research Center, Yorktown Heights, New York, NY 10598, USA; E-Mail: andot@us.ibm.com; Tel.: +1-914-945-1738; Fax: +1-914-945-4184

**Keywords:** high-κ, metal gate, scavenging, higher-κ, EOT, MOSFET

## Abstract

Current status and challenges of aggressive equivalent-oxide-thickness (EOT) scaling of high-κ gate dielectrics via higher-κ (>20) materials and interfacial layer (IL) scavenging techniques are reviewed. La-based higher-κ materials show aggressive EOT scaling (0.5–0.8 nm), but with effective workfunction (EWF) values suitable only for n-type field-effect-transistor (FET). Further exploration for p-type FET-compatible higher-κ materials is needed. Meanwhile, IL scavenging is a promising approach to extend Hf-based high-κ dielectrics to future nodes. Remote IL scavenging techniques enable EOT scaling below 0.5 nm. Mobility-EOT trends in the literature suggest that short-channel performance improvement is attainable with aggressive EOT scaling via IL scavenging or La-silicate formation. However, extreme IL scaling (e.g., zero-IL) is accompanied by loss of EWF control and with severe penalty in reliability. Therefore, highly precise IL thickness control in an ultra-thin IL regime (<0.5 nm) will be the key technology to satisfy both performance and reliability requirements for future CMOS devices.

## 1. Introduction

The rapid progress of complementary metal-oxide-semiconductor (CMOS) integrated circuit technology has been accomplished by a calculated reduction of the dimensions of the unit device in the circuit—a practice termed “scaling [1]”. Continued device scaling for future technology nodes requires reduction in equivalent oxide thickness (EOT) of gate dielectrics. Extendibility of the conventional SiO(N)/polycrystalline silicon (poly-Si) gate structure is challenged due to exponential increase in gate leakage currents [2]. As alternatives to SiO(N) gate dielectrics, much work has been done on the research of high permittivity (high-κ) materials [3]. After a decade-long search for the appropriate high-κ materials, the semiconductor industry has converged on Hf-based oxides, such as HfO_2_ or HfSi_x_O_y_, for the first generation CMOS products featuring high-κ gate dielectrics and metal gate electrodes (HKMG) [4,5]. The EOT for the first generation HKMG device is approximately 1.0 nm [4]. Continued L_g_ scaling for the 32 nm and beyond with a planer structure requires sub-nm EOT to suppress short-channel effects. Fully depleted device structures, such as FinFET or extremely thin SOI (ETSOI), improve short-channel control and thus relax the requirements for EOT scaling. However, the insertion point of such device architectures is expected to be the 22 nm and beyond and sub-nm EOT may be still required at those advanced technology nodes.

A typical HKMG stack structure contains a silicon oxide based interfacial layer (IL), a high-k dielectric, followed by a metal gate electrode. This system is equivalent to two capacitors connected in series. Thus, the total EOT of the HKMG stack can be expressed as follows.

(1) $$EOT=EOT_{IL}+EOT_{HK}$$

where EOT_IL_ and EOT_HK_ are contributions from the IL and high-κ layer, respectively. An apparent way to scale EOT_HK_ is to reduce the physical thickness of the high-κ layer, however, there is little room in this direction. It has been reported that a thick HfO_2_ layer causes significant degradations in carrier mobility and charge trapping both with gate-first [6] and gate-last [7] processes. Therefore, the first generation HKMG devices already employ the thinnest possible high-κ layer. This leaves us with three possible EOT scaling approaches: (1) Introduce a new high-κ material with k-value greater than that of HfO_2_; (2) Increase the k-value of IL; (3) Reduce the physical thickness of IL. In this paper, we review current status and challenges for each approach and discuss the EOT scaling strategy for future CMOS devices.

## 2. Higher-κ Materials

HfO_2_ is one of the most widely used high-κ materials, showing a k-value of approximately 20. Replacing HfO_2_ with materials having k-values greater than 20 (higher-κ) is a long term scaling solution. In pursuit of higher-k materials, the tradeoff between k-value and band gap needs to be taken into account. It is generally known that band gap values have a roughly inverse dependence on k-values (E_g_~k^−0.65^) [8]. Therefore, materials with too high k-values typically result in excessive direct tunneling currents and most work showing promising EOT-leakage current density (J_g_) characteristics has been achieved with k-value ranging from 20 to 30. Various groups have reported k-value increase in this range by stabilizing the higher-k phase (tetragonal or cubic) of HfO_2_ via doping of elements such as zirconium [9], yttrium [10], and silicon [11]. This is a practical means to attain a modest k-value increase, however, controllability of crystallinity in an ultra thin HfO_2_ thickness regime (<2 nm) has yet to be demonstrated. Other groups have suggested La-based higher-k materials such as La-Al-O or La-Lu-O [12]. These materials have recently demonstrated a lot of promise to outperform Hf-based high-κ on a device level [13,14,15,16].

**Figure 1:**
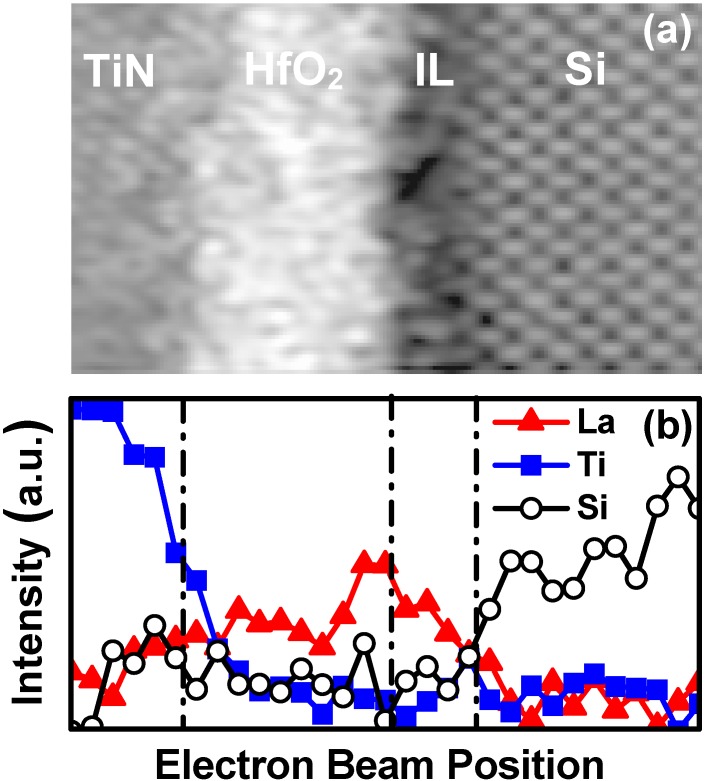
(**a**) Z-contrast image and (**b**) corresponding Electron Energy-Loss Spectroscopy (EELS) profiles (La, Ti, and Si) of the SiO_2_/HfO_2_/La_2_O_3_/TiN/poly-Si stack after a 1,000 °C anneal (after [19]).

A less disruptive way to increase the average k-value of the gate stack is to boost the k-value of the IL in conjunction with Hf-based oxides. A La_2_O_3_ cap on a Hf-based high-κ layer has been widely used to adjust the threshold voltage (V_t_) of n-type field-effect-transistors (nFET) [17]. Some portion of the La elements diffuse through the high-κ layer and form La-silicate IL after high temperature anneals [18]. This is an effective way to increase the k-value of SiO_2_-based IL. Figure 1(a,b) shows the Z-contrast image and Electron Energy-Loss Spectroscopy (EELS) profiles, respectively, obtained by Cs-corrected Scanning Transmission Electron Microscopy (STEM) for the SiO_2_/HfO_2_/La_2_O_3_/TiN/poly-Si stack after a 1,000 °C anneal (after [19]). As seen in Figure 1(b), formation of La-silicate in the IL can be precisely controlled by carefully optimizing the La_2_O_3_ layer thickness and the thermal budget, resulting in EOT of 0.58 nm. It was also reported that a single layer of La-silicate gate dielectric scales EOT to a similar value (0.62 nm) [20]. As a potential new alternative, it was recently shown that a thin epitaxial strontium oxide (SrO) interfacial layer prior to HfO_2_ deposition results in EOT of 0.50 nm [21] and 0.60 nm [22] for gate-last and gate-first integrations, respectively.

Table 1 summarizes recently reported IL k-boost and higher-κ data. EOT values less than 0.80 nm have been achieved with both approaches, however, the aggressive scaling is accompanied with effective workfunction (EWF) shift toward the Si conduction band minimum (CBM) in most cases. This trend is attributable to formation of electric dipoles at the SiO_2_/high-κ interface originating from lanthanide (e.g., La, Lu) or alkaline earth (e.g., Sr) metals [23,24,25]. Formation of such dipoles makes application of these materials to p-type field-effect-transistors (pFET) extremely difficult. Development of CMOS-compatible higher-κ materials is one of the biggest challenges to overcome. Unlike other lanthanide-based higher-κ materials, La-Al-O showed controllability of the EWF from the Si CBM to near the Si mid-gap with TiN electrodes by changing the La/(La+Al) atomic ratio [16,26,27]. This unique capability of La-Al-O may allow CMOS integration when combined with appropriate workfunction setting metals using gate-last process [28].

**Table 1:** Summary of silicon oxide based interfacial layer (IL) k-boost and higher-k data in literature. Equivalent oxide thickness (EOT), effective workfunction (EWF).

Materials	k-value	EOT (nm)	EWF with TiN electrode
La-silicate IL/(HfO_2_) [19,20]	-	0.58–0.62	~Si CBM
SrO IL/HfO_2_ [20,21]	-	0.50–0.60	~Si CBM
Y doped HfO_2_ [10]	27	-	-
Si doped HfO_2_ [11]	27	-	-
La-Lu-O [13]	23	0.58	~Si CBM
Y-La-Si-O [14]	-	0.77	~Si CBM
La_2_O_3_ [16]	-	0.62	~Si CBM
La-Al-O [15,16]	23–25	0.31–0.74	Si CBM~Si mid-gap

## 3. Interfacial Layer Scavenging Approach

As reviewed in Section 2, higher-κ materials are not mature enough to replace Hf-based oxide especially in terms of pFET EWF control although there has been great progress in EOT scaling in recent years (see Table 1). Considering the maturity of Hf-based high-κ gate dielectrics, scaling SiO_2_-based IL in conjunction with Hf-based oxides may be more practical in meeting the requirements for the 22 nm node and beyond. Several techniques going in this direction have been reported, such as IL scaling via scavenging reaction [19,29,30,31,32,33,34,35,36,37], cycle-by-cycle atomic layer deposition (ALD) and annealing of HfO_2_ [38], and advanced post deposition anneal for epitaxial HfO_2_ growth [39]. In this section, we mainly review the IL scavenging process since most device level learning is available in the literature with this approach.

### 3.1. Materials and Process Considerations

IL scaling via scavenging reaction has become a mainstream approach in recent years to realize EOT 0.5 nm and less. Several variations have been reported since Kim *et al.* found this phenomenon using a Ti overlayer on HfO_2_ or ZrO_2_ gate dielectrics [29]. Table 2 summarizes the key materials and process parameters for various IL scavenging processes in the literature. We have reviewed these IL scavenging processes from the following perspectives: (1) IL growth condition, (2) Choice of scavenging element, (3) Location of scavenging element, (4) Choice of high-κ material, (5) Maximum process temperature.

#### 3.1.1. IL Growth Condition

Ragnarsson *et al.* reported that EOT scaling via IL scavenging strongly depends on the growth condition of the initial IL [36]. In this work, room temperature chemical oxide ILs showed more EOT scaling compared to in-situ steam generation (ISSG) oxide ILs at 760–900 °C and thermal oxide ILs at 1,100 °C. It has been reported that IL scavenging reaction proceeds via decomposition of SiO_2_ and subsequent re-incorporation of Si atoms into the channel [29,34]. Since this is a reverse reaction of IL growth, a uniform SiO_2_ layer with low formation energy may be preferred as a starting IL to facilitate the scavenging reaction at later stages of process flow.

**Table 2:** Summary of IL scavenging processes in the literature.

Scavenging element	High-κ materials	Max temperature (°C)	EOT (nm)
Ti [29]	HfO_2_ or ZrO_2_	300	1.70
Ti [31]	HfO_2_	500	0.69
Hf [31]	HfO_2_	500	0.60
Hf [32]	HfO_2_	1020	0.59
TiN [36]	HfO_2_	1035	0.60
TiN [36]	HfO_2_/La-cap	1035	0.46
Doped TiN [19]	HfO_2_	1000	0.54
Doped TiN [19]	HfO_2_/La-cap	1000	0.42
Doped TiN [37]	HfO_2_	600	0.49

#### 3.1.2. Choice of Scavenging Element

**Figure 2:**
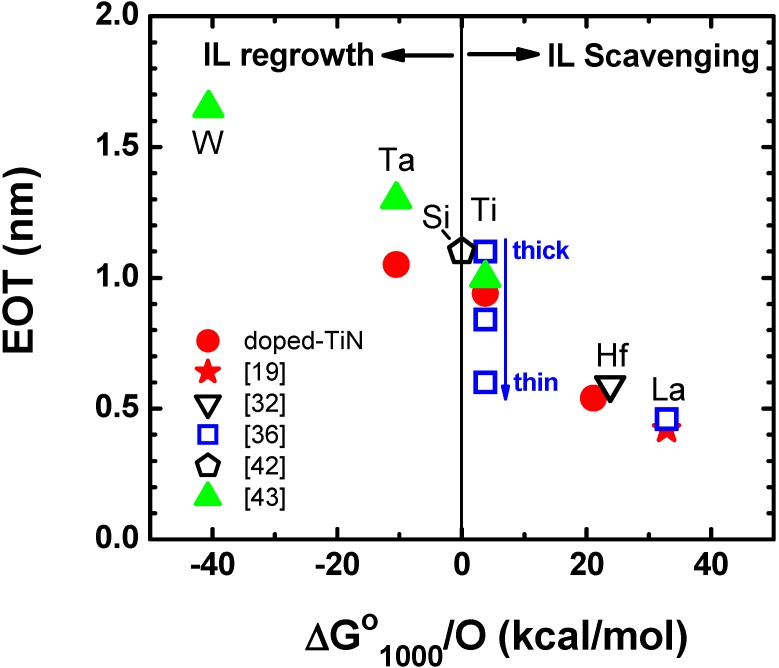
EOT of SiO_2_/HfO_2_ metal-inserted poly-Si stack (MIPS) structure as a function of ΔG^0^_1000_ per oxygen atom for scavenging element. TiN electrodes doped with various metals are compared with literature data (after [19,32,36,42,43]). The ΔG^0^_1000_ values are after [41].

Scavenging element is one of the most important factors for IL scavenging reaction, which affects the choice of other process parameters, such as location of scavenging element or maximum temperature in the subsequent processes. We found that the Gibbs free energy change at 1,000 K (ΔG^0^_1000_) of the following reaction serves as a guiding principle for the choice of scavenging element [19].

(2) $$Si+\frac{2}{y}M_{x}O_{y}\to \frac{2x}{y}M+SiO_{2}$$

where M is the scavenging element in the gate stack. When M with a positive ΔG^0^_1000_ value is doped in the gate stack, there is an energy gain for forming a metal oxide (M_x_O_y_) rather than maintaining SiO_2_, which is the driving force for the IL scavenging reaction. In the actual scavenging reaction, oxygen transport from SiO_2_ to M requires some energy. However, the experimental data suggest that oxygen diffusion through HfO_2_ or ZrO_2_ is quite rapid at elevated temperatures and this is not the rate-limiting step for the IL scavenging reaction [29,34]. The EOT trend for metal-inserted poly-Si stack (MIPS) [40] with SiO_2_/HfO_2_ dual-layer gate dielectrics in the literature is summarized in Figure 2 as a function of ΔG^0^_1000_ per oxygen atom (ΔG^0^_1000_/O) for the scavenging element. Here, the scavenging element is defined as the metal with the most positive ΔG^0^_1000_/O value (after [41]), existing in either gate dielectrics or workfunction-setting metal layers. For this comparison, data from the early days of gate-first development are also included (Poly-Si gate in [42]; W, TaN, TiN gates in [43]) to understand the IL scavenging process in a historical context.

As seen in Figure 2, EOT and ΔG^0^_1000_/O values show a very strong correlation. Poly-Si data point [42] serves as a reference since the ΔG^0^_1000_/O value is zero and neither IL regrowth nor scavenging is expected. Most thermally stable metals studied in the early phase of MGHK development (e.g., W, TaN in [43]) have negative ΔG^0^_1000_/O values, resulting in IL regrowth during high temperature activation anneals. On the other hand, TiN has a slightly positive ΔG^0^_1000_/O value, providing slight EOT scaling compared to Poly-Si in most cases (e.g., thick TiN in [36,43]). The near zero ΔG^0^_1000_/O value may be the main reason why TiN has been widely accepted as a baseline metal electrode throughout the industry. The neutral nature of TiN typically results in neither IL regrowth nor scavenging. However, it is possible to trigger IL scavenging by carefully optimizing the TiN thickness and employing an in-situ cap due to the slightly negative ΔG^0^_1000_/O value (e.g., thin TiN in [36]). In this case, a high thermal budget (>1,000 °C) is required since the driving force of the reaction is not very strong. In order to facilitate the reaction at lower temperatures, scavenging elements with much more positive ΔG^0^_1000_/O values are needed. Such elements, however, come with a severe penalty in thermal stability due to high reactivity with high-κ layers. In fact, early IL scavenging works employ pure transition metals with high ΔG^0^_1000_/O values at relatively low temperatures (300–500 °C [29,31]). Thus, direct capping of very strong scavenging metals may not be compatible with the state-of-the-art CMOS integration requiring higher process temperatures. To overcome this trade-off, we proposed doping of scavenging metals with high ΔG^0^_1000_/O values into a thermally stable TiN electrode [19,34]. This technique enables highly controllable IL scavenging for both gate-first (maximum process temperature: 1,000 °C) [19] and gate-last (maximum process temperature: 600 °C) [37] integrations.

As reviewed in this sub-section, the choice of appropriate scavenging metals, considering the thermal budget in downstream processes, is the key aspect of IL scavenging technique. We found that the ΔG^0^_1000_/O value for Equation (2) consistently explains the historical EOT trend and serves as a guiding principle for the material choice.

#### 3.1.3. Location of Scavenging Element

**Figure 3:**
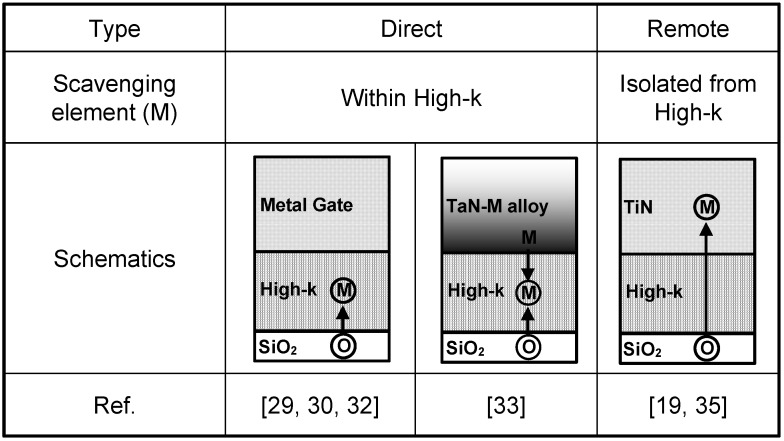
Schematics of direct- and remote- scavenging techniques in the literature.

**Figure 4:**
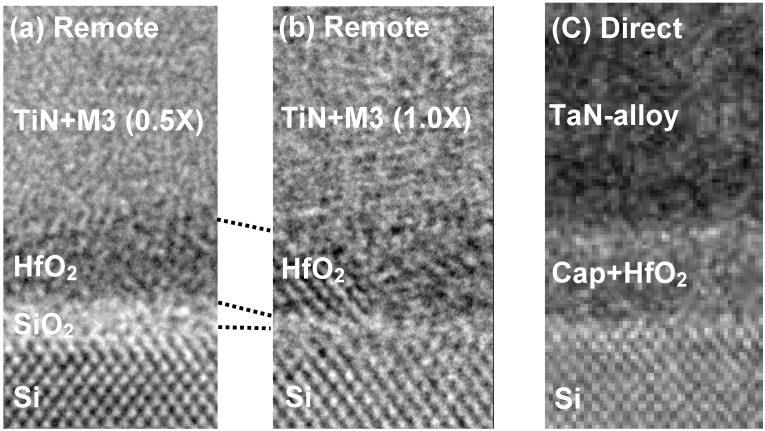
Cross-sectional transmission electron microscopy (TEM) images of the high-κ gate dielectrics and metal gate electrodes (HKMG) stacks for remote-scavenging with (**a**) medium scavenging metal dose; (**b**) high scavenging metal dose (after [19]); and (**c**) direct scavenging with TaN-alloy (after [33]).

In this sub-section, we have categorized various IL scavenging techniques into direct-scavenging and remote-scavenging based on the locations of the scavenging elements in the gate stacks. Figure 3 illustrates the difference between the two categories. Direct-scavenging schemes incorporate the scavenging elements within the high-κ layers, whereas the remote-scavenging schemes isolate the scavenging elements from the high-κ layers. Early IL scavenging works fall into the direct-scavenging category since the scavenging metals are incorporated within the high-κ layers either by direct capping [29,30] or by deposition of off-stoichiometric HfO_2_ [32]. Then, we reported IL scavenging using a TaN-alloy electrode [33]. This technique is still considered direct-scavenging since the scavenging metals diffuse out from the TaN-alloy during a high temperature anneal and end up in the HfO_2_ layer. The advantage of direct-scavenging is that it does not require such a strong driving force of the reaction (see ΔG^0^_1000_/O discussion in the previous sub-section). Therefore, a relatively weak scavenging metal such as Ti can cause aggressive EOT scaling [29,31]. The downsides of this approach include the following: (1) Effective workfunction change either by inherently low vacuum workfunction of scavenging metals or by formation of fixed charges and/or interface dipoles [29,31]; (2) Excessive carrier mobility degradation and leakage current increase [19]. One elegant way to perform direct-scavenging is to use Hf as a scavenging metal such that it becomes a part of the HfO_2_ gate dielectric after absorbing oxygen from the IL [30,32]. The degradation in device characteristics due to direct-scavenging can be mitigated to some extent in this way. We proposed the concept of remote-scavenging to completely overcome the aforementioned issues. By doping scavenging elements into a thermally stable TiN electrode with a certain distance from the high-κ/TiN interface, it is possible to cause IL scavenging reaction without out-diffusion of the scavenging elements into the high-κ layer. Such a process enables EOT scaling without extrinsic degradation in carrier mobility and leakage current and with no change in EWF [19]. Another way to carry out remote-scavenging is to employ a thin TiN electrode (~2 nm) with an in-situ Si cap to avoid oxidation from the ambient [35]. These remote-scavenging techniques demonstrated highly controllable EOT scaling and compatibility with advanced CMOS processing. Figure 4 shows cross-sectional transmission electron microscopy (TEM) images of the HKMG stacks for remote-scavenging with (a) medium scavenging metal dose, (b) high scavenging metal dose (after [19]), and (c) direct scavenging with TaN-alloy (after [33]), respectively. As shown in Figure 4(a,b), the final IL thickness is controllable by changing the scavenging metal dose. It is possible to completely eliminate the initial IL by both remote-scavenging and direct-scavenging (see Figure 4(b,c)).

#### 3.1.4. Choice of High-κ Material

**Figure 5:**
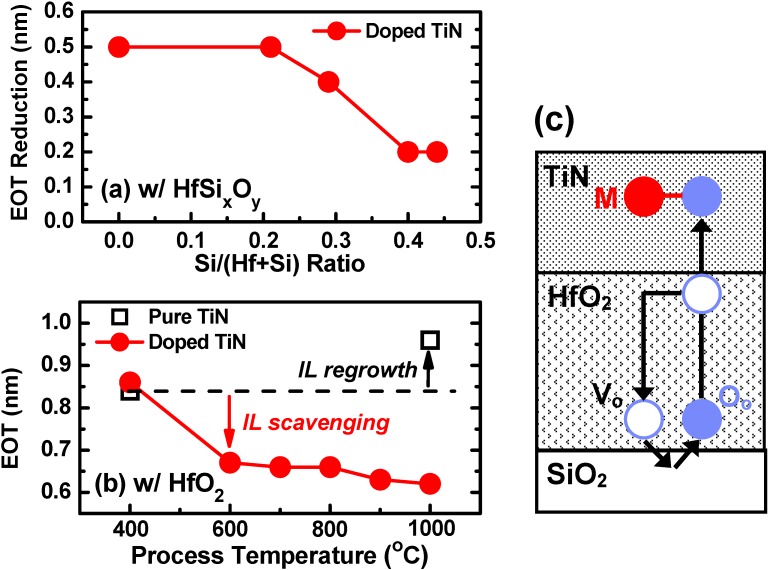
(**a**) EOT reduction via remote-scavenging from doped TiN electrodes as a function of Si/(Hf+Si) ratio in HfSi_x_O_y_ gate dielectrics. The EOT reduction is calculated from the difference between the doped TiN and the pure TiN; (**b**) EOT as a function of maximum process temperature for doped TiN and pure TiN using HfO_2_ gate dielectrics (after [34]); (**c**) Schematics of remote scavenging reaction. M, V_0_, and O_0_ represent the scavenging element, the oxygen vacancy in HfO_2_, and the oxygen atom in the lattice position of HfO_2_, respectively.

As shown in Table 2, most IL scavenging works used HfO_2_ or ZrO_2_ as gate dielectrics. In order to understand the role of high-κ layer on IL scavenging, we employed doped TiN electrodes on various compositions of HfSi_x_O_y_ layers with the Si/(Hf + Si) ratio ranging from 0.00 (HfO_2_) to 0.44 and fabricated metal-oxide-semiconductor (MOS) capacitors using gate-first process with a 1,000 °C activation anneal. Reference samples with pure TiN electrodes were also prepared. Figure 5(a) shows EOT reduction by remote IL scavenging (*i.e.*, EOT difference between the doped TiN and the pure TiN) as a function of the Si/(Hf + Si) ratio of HfSi_x_O_y_. The doping amount of the scavenging metal was chosen such that the initial IL (approximately 0.5 nm) is completely removed for HfO_2_. As the Si/(Hf + Si) ratio is increased, the EOT reduction is similar to that for HfO_2_ up to Si/(Hf + Si) ratio of 0.20 and then it starts to decrease. This trend indicates that ionic bonds (Hf-O) play a key role in remote-scavenging and the effect is hampered as the ratio of covalent bonds (Si-O) is increased. HfO_2_ and ZrO_2_ are very effective mediators of remote-scavenging from this perspective.

#### 3.1.5. Maximum Process Temperature

The discussion in Section 3.1.4 leads us to speculate that oxygen vacancies in ionic metal oxides contribute to remote-scavenging. We investigated the temperature dependence of remote-scavenging using HfO_2_ gate dielectrics in order to shed more light on the kinetics of the reaction. The maximum process temperature after the deposition of doped TiN electrodes was varied from 400 °C to 1,000 °C. As shown in Figure 5(b), most of the EOT scaling effect from the doped TiN already took place at 600 °C and little further change occurred with the 1,000 °C process (after [34]). In the case of pure TiN, a slight EOT increase was brought about by the 1,000 °C process. In contrast, the doped TiN served two purposes. One is the substantial EOT scaling via IL scavenging at the temperature between 400 and 600 °C. The other is the suppression of IL regrowth at the higher temperature. The former reaction coincides with the reported temperature at which oxygen vacancies in a HfO_2_ layer become mobile and reach a steady state for oxygen transport [44]. We speculate that the IL scavenging reaction proceeds in a remote way as the oxygen vacancies in the HfO_2_ layer act as mediators for oxygen transport from the IL to the TiN electrode. Thermal activation of the mobile oxygen vacancies in the HfO_2_ at 400–600 °C may be the threshold temperature for this reaction, provided that the driving force of the reaction (ΔG^0^_1000_/O) is large enough [34,37]. This reaction model is schematically illustrated in Figure 5(c). When the driving force of the reaction is small (e.g., TiN), oxygen scavenging itself becomes the rate-limiting step and much higher thermal budget (1,000 °C or higher) is required [36]. Thus, the required thermal budget for remote-scavenging strongly depends on the choice of scavenging element.

As reviewed in this Section, (1) IL growth condition; (2) Choice of scavenging element; (3) Location of scavenging element; (4) Choice of high-κ material; (5) Maximum process temperature, are the key considerations for IL scavenging. Careful materials and process design suited for the overall integration scheme (e.g., gate-first or gate-last) is indispensable for the implementation of IL scavenging in state-of-the-art CMOS devices.

### 3.2. EOT Scaling and Gate Leakage Current

**Figure 6:**
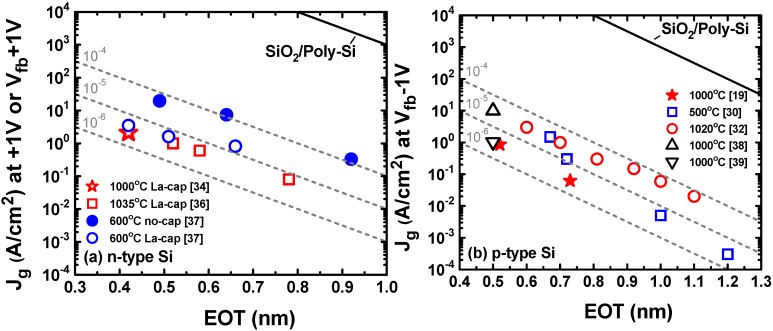
(**a**) EOT-J_g_ characteristics for n-type metal-oxide-semiconductor (MOS) capacitors with (SiO_2_)/HfO_2_/(La-cap) gate dielectrics in literature. J_g_ is defined at V_g_ = flatband voltage (V_fb_) + 1 V for [34,37] and at V_g_ = 1 V for [36]; (**b**) EOT-J_g_ characteristics for p-type MOS capacitors with (SiO_2_)/HfO_2_ gate dielectrics in literature. J_g_ is defined at V_g_ = V_fb_ − 1 V for [19,30,32,38,39]. The slopes for ideal SiO_2_ scaling are shown in broken lines with the magnitude in J_g_ reduction from SiO_2_/Poly-Si stacks in both plots. The maximum process temperatures are indicated in the legends.

The polarity of gate leakage current through SiO_2_/HfO_2_ dual-layer gate dielectrics depends on the gate bias polarity and substrate doping type. For typical SiO_2_/HfO_2_ stacks, electron injection from the Si conduction band is the dominant tunneling current component under positive gate bias (n-type substrate in accumulation), whereas hole injection from the Si valence band is the majority current under negative gate bias (p-type substrate in accumulation) [45,46]. Therefore, we compared the EOT-J_g_ characteristics of IL scavenging both for n-type and p-type MOS capacitors in accumulation. For this comparison, only direct-scavenging using Hf [30,32] and remote-scavenging [19,34,36,37] are included to rule out the extrinsic degradation in J_g_ as discussed in Section 3.1.3. In addition, these IL scavenging data are compared with zero-IL HfO_2_ stacks obtained by other methods (cycle-by-cycle ALD and annealing of HfO_2_ [38] and advanced post deposition anneal for epitaxial HfO_2_ growth [39]). Multiple data points from the same reference are obtained by changing the IL thickness with a fixed HfO_2_ thickness. Figure 6(a,b) show the EOT-J_g_ characteristics in literature for n-type MOS capacitors and p-type MOS capacitors, respectively. The slopes for ideal SiO_2_ scaling (10× increase in J_g_ per 0.2 nm scaling) are included in both plots as a guide. It should be noted that the experimental slopes for IL scavenging follow the ideal SiO_2_ scaling in a wide EOT range on both bias conditions irrespective of the maximum process temperatures (500–600 °C in [30,37], 1,000–1,035 °C in [19,32,36]). This suggests that optimized IL scavenging techniques can avoid extrinsic J_g_ degradation for both nFET and pFET either with gate-first or gate-last integration. As shown in Figure 6(a), La-caps help in reducing J_g_ with little EOT penalty. Thus, the most aggressive EOT scaling (0.42 nm) was obtained by IL scavenging in conjunction with La-caps [34]. Comparison between IL scavenging techniques and other methods [38,39] is shown in Figure 6(b). In all cases, EOT scaling down to 0.50 nm was achieved using pure HfO_2_, indicating that the ultimate scaling point (zero-IL) does not depend on the method. However, IL scavenging techniques do offer an advantage over other methods in adjusting the IL thickness anywhere between the as-grown thickness and zero on the ideal SiO_2_ scaling trend in EOT-J_g_ characteristics [19,32,36,37]. Such controllability of IL thickness is extremely important when we consider EWF control and device reliability in conjunction with aggressive EOT scaling as we review in Section 3.3 and Section 3.4.

### 3.3. Effective Work Function Control

As discussed in Section 3.1.3, remote-scavenging process per se does not change the EWF of the gate stack, which makes this technique applicable to both nFET and pFET if the EWF is independently tuned by other knobs. In the case of gate-first process, La and Al cap layers on Hf-based high-κ gate dielectrics are widely practiced to control the EWF for nFET and pFET, respectively [17,40]. The origin of the EWF change has been identified to be the electric dipoles located at the SiO_2_/high-κ interface, however, the exact mechanism of the interface dipole formation is still controversial [25,27,47,48].

**Figure 7:**
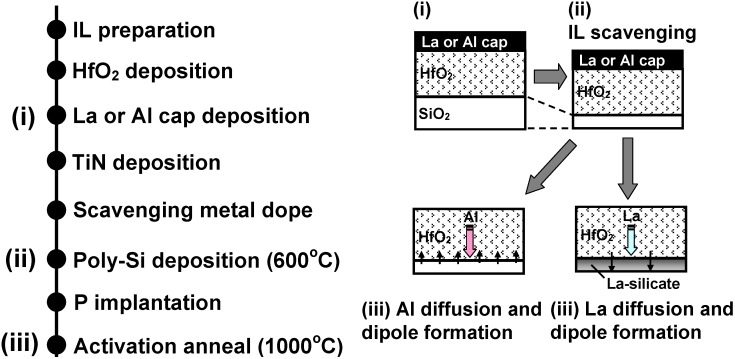
Process flow for MOS capacitors using La-(Al-) caps in conjunction with remote IL scavenging. The change in the gate dielectrics at the key steps is illustrated in the schematics. The corresponding electrical data are shown in Figure 8.

**Figure 8:**
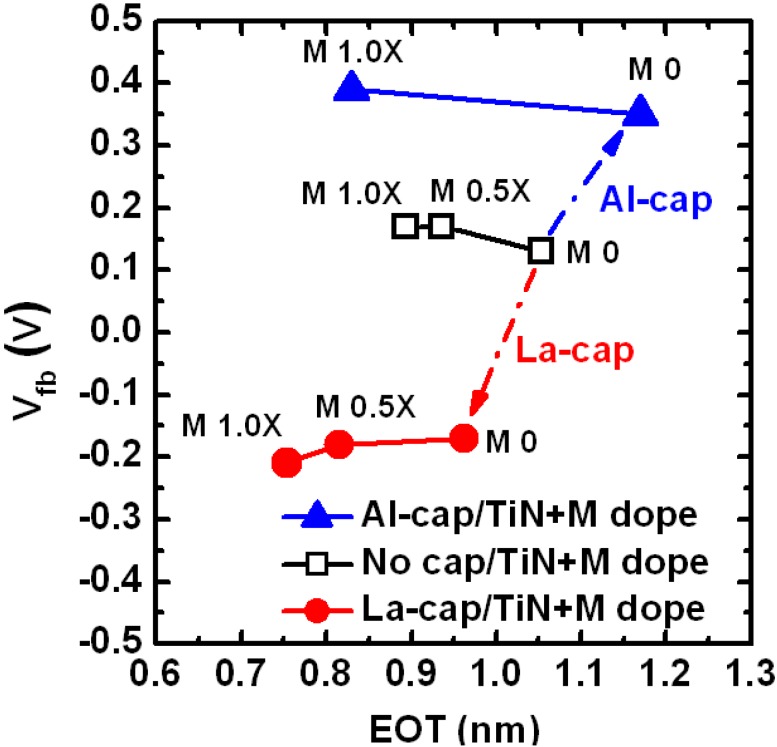
EOT-V_fb_ plot for MOS capacitors prepared with process flow in Figure 7. The V_fb_ values were tuned toward a negative (positive) direction using La-(Al-) caps. The EOT values were controlled by changing the amount of scavenging metal (M) dose in the TiN electrodes.

When the La-(Al-) cap and remote-scavenging techniques are simultaneously employed, we need to consider the kinetics of both reactions. It was reported that the La-(Al-) cap requires at least 800 °C to diffuse through the HfO_2_ and reach the SiO_2_ IL [18,25]. After the La (Al) atoms reach the SiO_2_ IL, thermal budget as low as 300 °C is enough to activate the La- (Al-) induced dipoles [27]. Therefore, the diffusion of La (Al) atoms is the limiting factor for the interface dipole formation. On the other hand, the remote scavenging reaction can happen in a much lower temperature range (400–600 °C) if a sufficiently strong scavenging element is used as discussed in Section 3.1.5. Figure 7 shows the process flow for the MOS capacitors using La-(Al-) caps in conjunction with remote IL scavenging. Due to the difference of kinetics between the remote-scavenging and the interface dipole formation, the chronological order of the reactions is as follows: (1) The remote-scavenging happens during the Poly-Si deposition typically at 500–600 °C; (2) La (Al) atoms diffuse through the HfO_2_ layer during the activation anneal typically at 1,000 °C or higher; (3) La-(Al-) induced dipole layers form at the bottom IL, as schematically illustrated in Figure 7. The corresponding EOT-V_fb_ data are shown in Figure 8 (after [19]). The IL thickness was varied by changing the scavenging metal (M) dose. As seen in Figure 8, V_fb_ tuning by La-(Al-) caps and EOT scaling by remote-scavenging are compatible and can be independently controlled. Thus, the chronological order of the reactions enabled IL scavenging without disruption of the interface dipole formation. However, the EWF controllability by the La-(Al) caps is lost when the SiO_2_ IL is completely scavenged [49]. In addition, it was reported that complete elimination of IL from a SiO_2_/HfO_2_ stack causes disappearance of the interface dipoles originating from Si-O-Hf bonds, resulting in disruptive shifts of V_fb_ [50]. Therefore, leaving an ultra-thin SiO_2_ IL after scavenging may be the key to avoid a disruptive change of EWF and to allow tuning by interface dipoles for CMOS integration using gate-first process.

In the case of gate-last process, EWF tuning for nFET and pFET is typically achieved by employing dual metal gates with appropriate work functions [4,51]. Since gate-last process does not rely on interface dipole for EWF adjustment, IL scavenging should not play a direct role on EWF setting. However, it was recently reported that the EWFs of gate-last pFET stacks start to roll-off when EOT is scaled below 0.8 nm [52,53]. Similar EWF roll-off behaviors were initially reported for gate-first process at a much thicker EOT regime (~3 nm) [54,55], motivating many people to move to gate-last process to realize low threshold voltage pFETs. We found that the same phenomenon takes place for gate-last process as the SiO_2_ IL thickness scales below 0.4 nm [37]. Based on our direct tunneling current analysis of SiO_2_/HfO_2_ dual-layer stacks using the transfer-matrix-approach [46,56], it is suggested that the band offsets for the SiO_2_ and HfO_2_ layers are reduced in the submonolayer IL regime, which may be related to the existence of a suboxide transition region at the Si/SiO_2_ interface (~0.3 nm) [57]. Such degradation in film quality may facilitate oxygen vacancy generation in the HfO_2_ layer [54,58] and/or the SiO_2_ IL [55], resulting in the unfavorable EWF shift for pFET. Thus, leaving an ultra-thin and robust SiO_2_ IL after scavenging is indispensable for EWF control of gate-last process as well.

### 3.4. Implications for Reliability and Carrier Mobility

**Figure 9:**
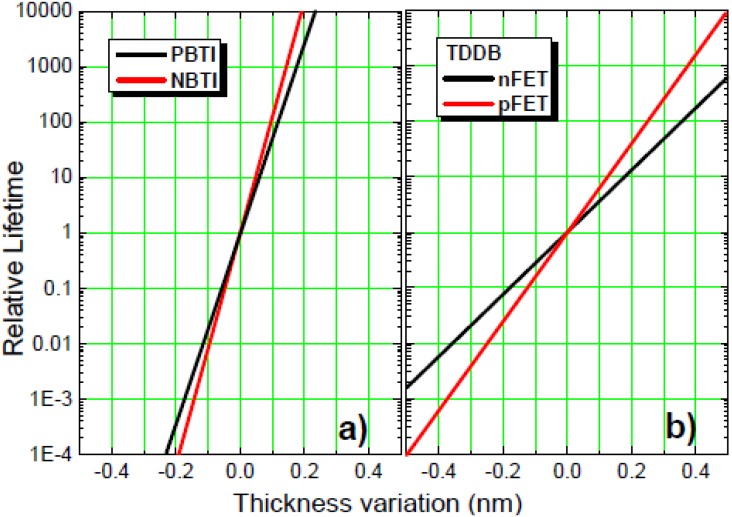
(**a**) Relative change of positive bias temperature instability (PBTI) and negative bias temperature instability (NBTI) lifetime with gate oxide thickness change via IL scavenging; (**b**) Relative change of time dependent dielectric breakdown (TDDB) lifetime for nFET and pFET with gate oxide thickness change via IL scavenging (after [61]).

Other device parameters requiring close attention with aggressive EOT scaling are reliability and carrier mobility. We investigated impacts of remote IL scavenging on reliability using constant voltage stress (CVS) and voltage ramp stress (VRS) [59,60]. SiO_2_/HfO_2_ dual-layer stacks with varying IL thicknesses were prepared in both gate-first and gate-last processes for this analysis. The change in the device lifetimes, including positive bias temperature instability (PBTI), negative bias temperature instability (NBTI), and time dependent dielectric breakdown (TDDB), are estimated and summarized in Figure 9 (after [61]). Note that the estimated lifetime trends for IL scaling are similar for gate-first and gate-last processes, indicating that these trends arise from the fundamental materials properties of the SiO_2_/HfO_2_ dual-layer stacks and do not depend much on the fabrication method [61]. As shown in Figure 9, the BTI lifetimes are predicted to decrease by 50–100× for every 0.1 nm of IL scaling. Drastic lifetime reductions also occur for TDDB. Therefore, calculated IL scaling, considering reliability requirements, is needed for future CMOS nodes.

**Figure 10:**
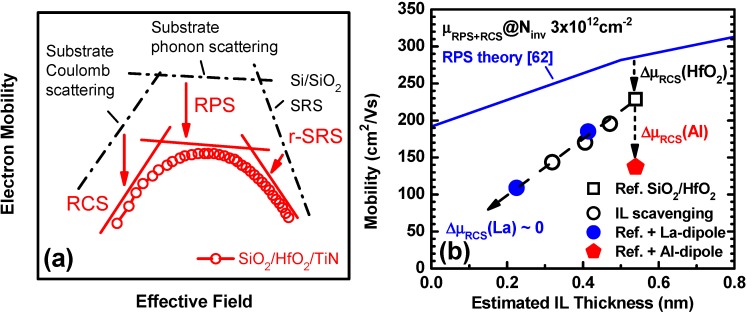
(**a**) Impacts of SiO_2_/HfO_2_/TiN stack on electron mobilities at different E_eff_. The red curve is obtained from a typical SiO_2_/HfO_2_/TiN stack prepared with gate-first process; (**b**) Electron mobility limited by RPS and RCS (μ_RPS + RCS_) at 300 K as a function of estimated IL thickness at N_inv_ of 3 × 10^12^ cm^−2^ (after [34]). The data for remote IL scavenging and La-(Al-) induced dipoles are included. The simulated dependence from the RPS theory [62] with ion impurity correction is also shown.

The implication of IL scaling for carrier mobility is another important factor for device performance. Although a great deal of progress has been made across the community, electron mobility values for HKMG stacks typically fall below the trend for conventional SiO(N)/Poly-Si stacks. The degradation is attributed to intrinsic properties of high-κ, such as soft optical phonons [62], fixed charges [63,64,65], surface roughness [19,64], and interface dipoles [66]. Figure 10 (a) illustrates impacts of a SiO_2_/HfO_2_/TiN stack on electron mobilities at different effective fields (E_eff_). In the case of ideal thermal oxide gate dielectrics, the electron mobility is limited by Coulomb scattering from the substrate impurities (low E_eff_), phonon scattering from the Si substrate (middle E_eff_), and surface roughness scattering from the Si/SiO_2_ interface (high E_eff_) [67]. It has been reported by many researchers that introduction of high-κ dielectrics brings about additional degradation in each E_eff_ regime. In the low E_eff_ regime, fixed charges in the high-κ layer or interface dipoles localized at the IL/high-κ interface cause remote Coulomb scattering (RCS) [63,64,65,66]. In the middle E_eff_ regime, Fischetti *et al.* predicted that soft optical phonons in high-κ layers can couple with carrier electrons, resulting in remote phonon scattering (RPS) [62]. In the high E_eff_ regime, either the fluctuation of permittivity of high-κ layers or the surface roughness at the IL/high-κ interface can cause remote surface roughness scattering (r-SRS) [19,64]. All of these additional scattering mechanisms are predicted to show strong dependences on the IL thickness. Therefore we studied the impacts of remote IL scavenging and La-(Al-) induced IL modification on electron mobility.

We performed low temperature mobility measurements and decoupled the SRS component from the RPS and RCS components [19,34]. The RPS- and RCS-limited mobility (μ_RPS+RCS_) at inversion carrier density (N_inv_) 3 × 10^12^ cm^−2^ as a function of estimated IL thickness is shown in Figure 10(b) (after [34]). Simulated IL dependence from the RPS theory [62] is shown in blue line. The experimental data for remote IL scavenging delineate a straight tradeoff line, which is slightly steeper and shifted downwards from the prediction based on the RPS theory. The deviation is attributable to the additional RCS from the fixed charges [65] and/or interface dipoles [66] at the SiO_2_/HfO_2_ interface. It is interesting to note that the trend line for the La-induced dipole is identical to that of the intrinsic IL scaling, indicating no additional RCS. In contrast, the Al-induced dipole brought about additional RCS at a fixed IL thickness. This difference may originate from the silicate forming nature of La [68], resulting in long-range and low density electric dipoles [27]. Thus, La-silicate IL is a promising EOT scaling knob (see Section 2) with no additional mobility degradation, although it is only suitable for nFET due to the EWF shift toward the Si CBM (see Table 1).

**Figure 11:**
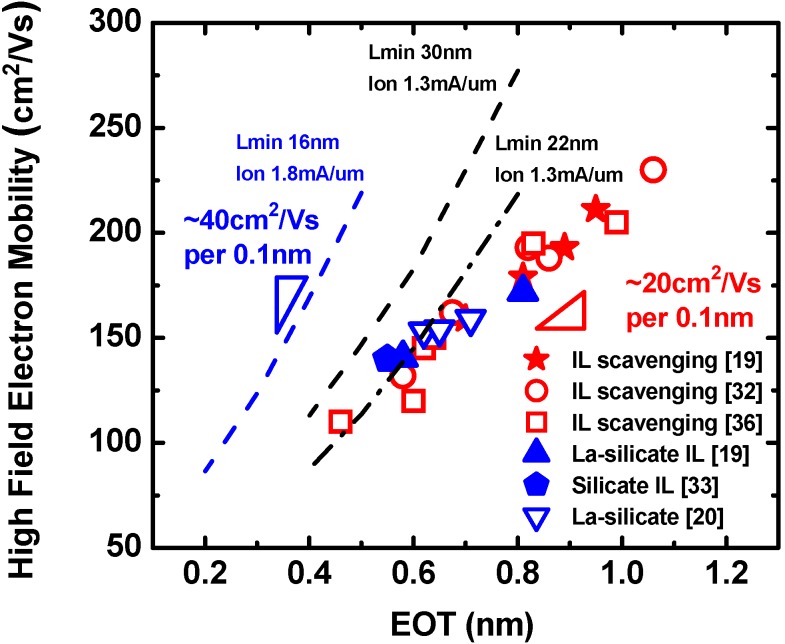
High field electron mobility as a function of EOT from literature data including remote IL scavenging [19,36], direct IL scavenging using Hf [32], La-silicate IL with HfO_2_ [19,33], and single-layer La-silicate [20]. The mobility values are taken at N_inv_ = 1 × 10^13^ cm^−2^ in [36] and at E_eff_ = 1 MV/cm in all other work. Simulated contour lines providing the same I_on_ at L_min_ 16, 22, and 30 nm are shown for comparison (after [75]).

On the other hand, the mobility degradation in the high E_eff_ regime is solely limited by SRS [19,34]. Li and Ma predicted that roughness at the remote interface from the channel can degrade the carrier mobility depending on the oxide thickness (T_IL_) and the average deviation of T_IL_(ΔT_IL_) [69]. We speculate that r-SRS from the SiO_2_/HfO_2_ interface is the origin of the mobility degradation, resulting in a strong IL-thickness dependence of high field electron mobility. Figure 11 shows high field electron mobility as a function of EOT from literature data including remote IL scavenging [19,36], direct IL scavenging using Hf [32], La-silicate IL with HfO_2_ [19,33], and single-layer La-silicate [20]. It should be noted that all data fall on the same line with a slope of approximately 20 cm^2^/Vs per 0.1 nm scaling, showing the universality of our IL thickness dependent mobility model in the ultra-thin EOT regime [19,34]. Moreover, negligible impact of La-induced dipoles on mobility is evidenced by the convergence of the IL scavenging trend and the La-silicate trend.

### 3.5. EOT Scaling Strategy

Understanding of correlation between low-field mobility (μ) and high-field carrier velocity (*v*), which is more directly related to drive current, becomes more and more important in the state-of-the-art CMOS devices. The correlation depends on the gate length (L_g_) [70]. In long-channel MOSFETs, μ is the sole factor in determining *v*. As velocity saturation phenomenon begins to occur in short-channel MOSFETs, μ dependence of *v* becomes weaker [71]. As the L_g_ shrinks further, the carrier transport eventually reaches a full-ballistic regime where μ loses its meaning and *v* is determined solely by injection velocity [72]. Modern MOSFETs, however, are considered to operate in a quasi-ballistic transport regime [73] where μ still plays an important role via backscattering ratio for carriers injected from source to channel [74]. Therefore, a higher μ is still preferred at a given EOT for the near-term CMOS scaling until full-ballistic operation is realized.

Ideally, gate dielectric scaling should be achieved without carrier mobility degradation. As reviewed in Section 3.4., this is fundamentally not possible if we employ IL scaling. The alternative is to maintain sufficiently thick IL and to introduce a higher-κ material. The mobility-EOT trade off may be mitigated in this way, however, materials reaching the maturity of Hf-based high-κ have not been identified yet as reviewed in Section 2. Therefore, EOT scaling with some compromise of carrier mobility is the only available scaling approach. In this regard, Tatsumura *et al.* predicted a mobility-EOT relationship providing the same drive current (I_on_) at a given L_min_ (L_g_ defined by off-state leakage current) by considering both mobility degradation and short-channel effects suppression from EOT scaling [75]. The comparison between the experimental data in the literature and the extracted contour lines from [75] is shown in Figure 11. The mobility-EOT slope for IL scavenging and La-silicate IL (~20 cm^2^/Vs per 0.1 nm) is shallower than the breakeven relationship for L_min_ ≤ 30 nm (~40 cm^2^/Vs per 0.1 nm). This indicates that it is indeed possible to improve the short-channel device performance by employing IL scaling in conjunction with aggressive L_g_ scaling in future nodes albeit with some electron mobility degradation. The key to realize performance improvement going in this direction is to follow the ideal IL scaling trend by optimizing the IL scavenging process and/or by employing a silicate IL which does not cause additional carrier scattering (e.g., La-silicate).

Although, IL scaling can provide performance improvement, extreme IL scaling (e.g., zero-IL) ends up with loss of EWF control both in gate-first and gate-last processes (see Section 3.3) and with severe penalty in reliability (see Section 3.4). Therefore, a highly controllable IL scavenging technique which can adjust the IL thickness to the optimum point where both performance and reliability requirements are satisfied is required. Such IL thickness control has been demonstrated for remote IL scavenging by changing the scavenging metal dose in the TiN electrodes [19,34,37] or by adjusting the TiN thickness with the in-situ Si cap [35,36]. These are encouraging techniques to extend Hf-based high-κ dielectrics toward the end of the CMOS roadmap.

## 4. Conclusions

EOT scaling into the sub-nm regime has been accomplished by various approaches. La-based higher-κ materials and La-silicate IL with HfO_2_ showed aggressive EOT values (0.5–0.8 nm), but with large EWF shifts toward the Si CBM, limiting their application to nFET. Further exploration for pFET-compatible higher-κ materials is needed. Meanwhile, IL scavenging is a promising approach to extend Hf-based high-κ dielectrics to future nodes. Remote-scavenging techniques enable EOT scaling below 0.5 nm. The key considerations for IL scavenging are (1) IL growth condition; (2) Choice of scavenging element; (3) Location of scavenging element; (4) Choice of high-κ material and (5) Maximum process temperature. Careful materials and process choice based on these considerations is indispensable. Mobility-EOT trends in the literature suggest that short-channel performance improvement is attainable with aggressive EOT scaling via IL scavenging or La-silicate formation. However, extreme IL scaling is accompanied with loss of EWF control and with severe penalty in reliability. Therefore, highly precise IL thickness control in an ultra-thin IL regime (<0.5 nm) will be the key technology to satisfy both performance and reliability requirements for future CMOS devices.
